# Mechanism of carvedilol induced action potential and calcium alternans

**DOI:** 10.1080/19336950.2022.2055521

**Published:** 2022-05-02

**Authors:** Elizabeth Martinez-Hernandez, Giedrius Kanaporis, Lothar A. Blatter

**Affiliations:** Department of Physiology & Biophysics, Rush University Medical Center, Chicago, Illinois, USA

**Keywords:** Action potential, alternans, atrium, calcium transient, carvedilol

## Abstract

Carvedilol is a nonspecific β-blocker clinically used for the treatment of cardiovascular diseases but has also been shown to have profound effects on excitation-contraction coupling and Ca signaling at the cellular level. We investigate the mechanism by which carvedilol facilitates Ca transient (CaT) and action potential duration (APD) alternans in rabbit atrial myocytes. Carvedilol lowered the frequency threshold for pacing-induced CaT alternans and facilitated alternans in a concentration-dependent manner. Carvedilol prolonged the sarcoplasmic reticulum (SR) Ca release refractoriness by significantly increasing the time constant τ of recovery of SR Ca release; however, no changes in L-type calcium current recovery from inactivation or SR Ca load were found after carvedilol treatment. Carvedilol enhanced the degree of APD alternans nearly two-fold. Carvedilol slowed the APD restitution kinetics and steepened the APD restitution curve at the pacing frequency (2 Hz) where alternans were elicited. No effect on the CaT or APD alternans ratios was observed in experiments with a different β-blocker (metoprolol), excluding the possibility that the carvedilol effect on CaT and APD alternans was determined by its β-blocking properties. These data suggest that carvedilol contributes to the generation of CaT and APD alternans in atrial myocytes by modulating the restitution of CaT and APD.

## Introduction

Cardiac alternans is a periodic beat-to-beat alternation in action potential duration (APD or electrical alternans), cytosolic Ca ([Ca]_i_) transient amplitude (CaT alternans) or contraction strength (mechanical alternans). Cardiac alternans plays a significant role in the development of cardiac arrhythmia, including atrial fibrillation (AF), and sudden cardiac death [1,2]. Cardiac alternans is multifactorial; however, the interplay between [Ca]_i_ and membrane voltage (V_m_) is a key factor in its onset and development. [Ca]_i_ and V_m_ regulation are bi-directionally coupled. V_m_ affects [Ca]_i_ by regulating voltage-dependent ion conductances, including L-type Ca channels, and modulates cardiac excitation-contraction coupling (ECC) and Ca induced Ca release (CICR) from the sarcoplasmic reticulum (SR) Ca store. At the same time, [Ca]_i_ modulates V_m_ and AP morphology through Ca-dependent membrane conductances, such as the sodium-calcium exchanger (NCX), Ca-activated Cl channels, and L-type Ca current (I_Ca_) [3,4]. The observation that CaT alternans can occur in the presence or absence of APD alternans puts cellular Ca handling at the center of cardiac alternans mechanism [4–7], although experimental studies have attributed cardiac alternans to both a primary disturbance of V_m_ and [Ca]_i_ regulation [8–11].

β-adrenergic receptor antagonists (β-blockers), such as carvedilol, are clinically used for hypertension and heart failure treatment to reduce arrhythmia risk, including AF, and to prevent sudden cardiac death [12–14]. We have shown previously that at the cellular level, carvedilol causes ECC failures by blocking voltage-gated Na and Ca channels in atrial myocytes [15]. Carvedilol has also been described to significantly reduce ryanodine receptor (RyR) SR Ca release channel mean open time and to a lesser extent its open probability (P_o_) which in turn decreases the propensity of spontaneous arrhythmogenic RyR Ca release such as Ca waves [15,16]. Inhibition of RyR function modifies SR Ca release refractoriness and therefore changes [Ca]_i_ and susceptibility to cardiac alternans [5,17–23]. It has been reported that carvedilol facilitates CaT alternans [23] in hearts carrying a suppression-of-function RyR mutation (E4872Q), however this has not been described for normal cardiac tissue, including the atria. Here, using a combination of current- and voltage-clamp recordings, and simultaneous optical measurements of APs and [Ca]_i_, we demonstrate that carvedilol induces CaT and APD alternans in rabbit atrial myocytes by altering AP restitution and prolonging SR Ca release refractoriness.

## Materials and methods

### Myocyte isolation

Atrial myocytes were isolated from male New Zealand white rabbits (2.5 kg; 41 rabbits; Envigo, Indianapolis, IN). Rabbits were anesthetized with an intravenous injection of pentobarbital sodium (Euthasol; 50 mg/kg) together with heparin (1,000 IU/kg). Hearts were excised and mounted on a Langendorff apparatus and retrogradely perfused for 5 min with nominally Ca-free Tyrode solution (in mM): 140 NaCl, 4 KCl, 1 MgCl_2_, 10 D-glucose, and 10 HEPES, heparin (1,000 IU/kg); pH 7.4 with NaOH. All chemicals and reagents were from MilliporeSigma (St. Louis, MO, USA) unless otherwise stated. Ca-free Tyrode perfusion was followed by perfusion with minimal essential medium Eagle (MEM) solution containing 20 µM Ca and 22.5 µg/ml Liberase TH (Roche Applied Science, Indianapolis, IN) for ~30 min at 37°C. The left atrium was removed and digested for an additional 5 min in the enzyme solution at 37°C. Digested tissue was then minced, filtered, and washed in MEM solution containing 50 µM Ca and 10 mg/ml bovine serum albumin. Isolated cells were kept in MEM solution with 50 µM Ca at room temperature (22–24°C) until experimentation. All aspects of animal husbandry, animal handling, anesthesia, surgery, and euthanasia were fully approved by the Institutional Animal Care and Use Committee (IACUC) of Rush University Chicago.

### Patch clamp experiments

V_m_ and I_Ca_ were recorded from single atrial myocytes in the whole cell ruptured patch clamp configuration using an Axopatch 200B patch-clamp amplifier, the Axon Digidata 1550B interface, and pCLAMP 11 software (Molecular Devices, Sunnyvale, CA). Current and voltage clamp recordings were low pass filtered at 5 kHz and digitized at 10 kHz. Patch pipettes (1.5–3 MΩ filled with internal solution) were pulled from borosilicate glass capillaries (WPI, Sarasota, FL, USA) with a horizontal puller (model P-97; Sutter Instruments, Novato, CA).

Current-clamp measurements were conducted in standard Tyrode solution containing (in mM) 135 NaCl, 4 KCl, 2 CaCl_2_, 1 MgCl_2_, 10 D-glucose, and 10 HEPES (pH 7.4 with NaOH). The pipette solution contained (in mM) 130 K-glutamate, 5 NaCl, 15 KCl, 0.33 MgCl_2_, 4 MgATP, and 10 HEPES (pH 7.2 with KOH). APs were evoked by 5 ms stimulation pulses of a magnitude ~1.5 times higher than AP activation threshold, and cells were stimulated at 1 or 2 Hz. To measure AP restitution, a double pulse (S1-S2) protocol was used (Figure 4A). The diastolic interval (DI) between S1 and S2 ranged from 250 to 1,250 ms. A train of steady-state APs was recorded for 15 s before applying the S1-S2 protocol. Experiments were conducted at room temperature (22–24°C).

**Figure 1.**
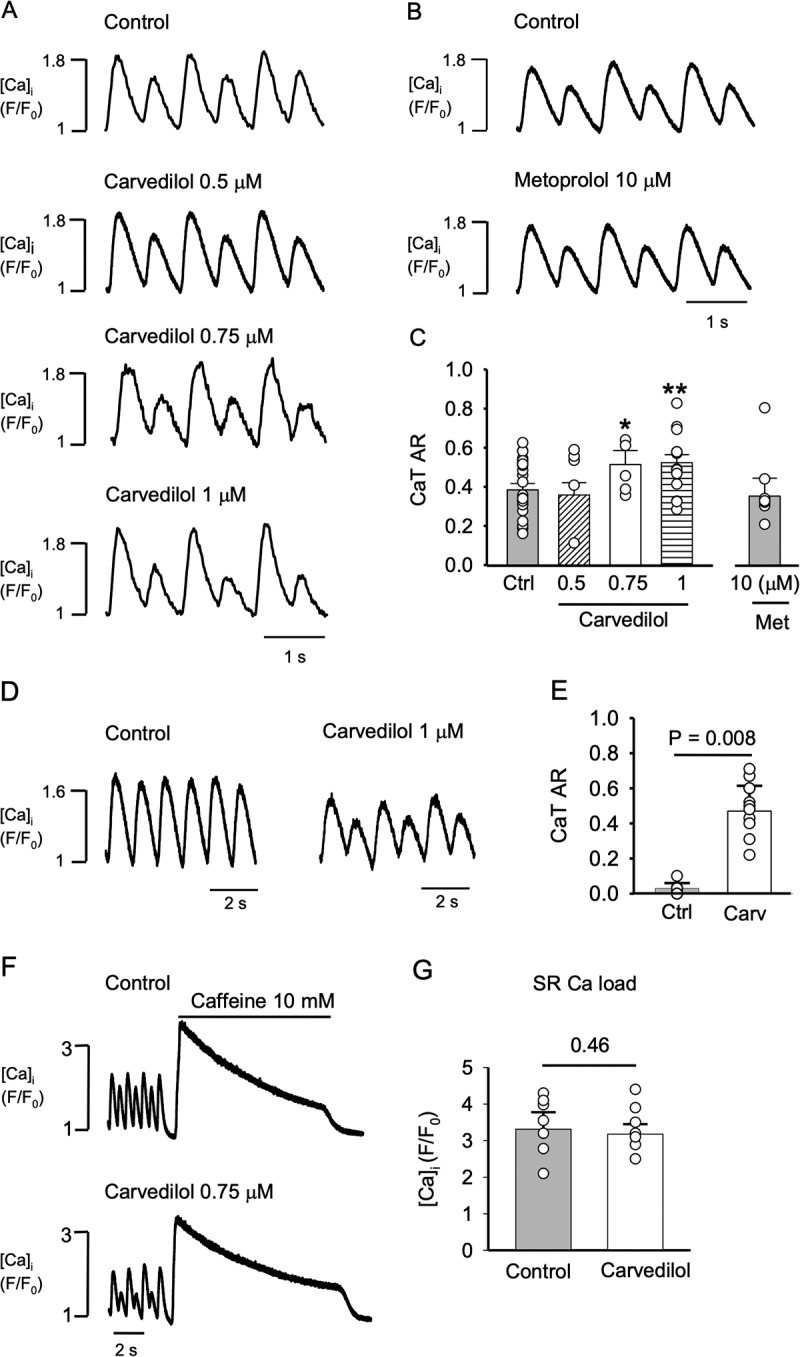
**Carvedilol increases CaT alternans susceptibility**. A, Representative traces of CaT alternans recorded in control and in presence of carvedilol at 0.5, 0.75 and 1 µM. B, CaT alternans in control and in presence of metoprolol (10 µM) as negative control. C, Mean CaT alternans ratio (AR) from control and carvedilol (0.5 µM: P = 0.31, n = 5; 0.75 µM: * P = 0.039, n = 11; 1 µM: ** P = 0.001, n = 14) or metoprolol (Met; 10 µM; P = 0.41, n = 7) treated atrial myocytes. All experiments are paired measurements of control and drug effect measured in the same cell and statistical analysis (paired t-test) was done against concentration specific controls. For presentation purpose only, all controls were pooled together. D, Representative CaT traces recorded in control and in presence of carvedilol (1 µM) at 1 Hz pacing rate. E, Mean CaT alternans ratio (AR) in control and 1 µM carvedilol (1 Hz; P = 0.008, n = 11). F, Carvedilol effect on SR Ca load was quantified as the amplitude of the CaT elicited by caffeine (10 mM) in control (top) and in the presence of 0.75 µM carvedilol (bottom). G, Average CaT amplitudes in response to caffeine (n = 7).

**Figure 2.**
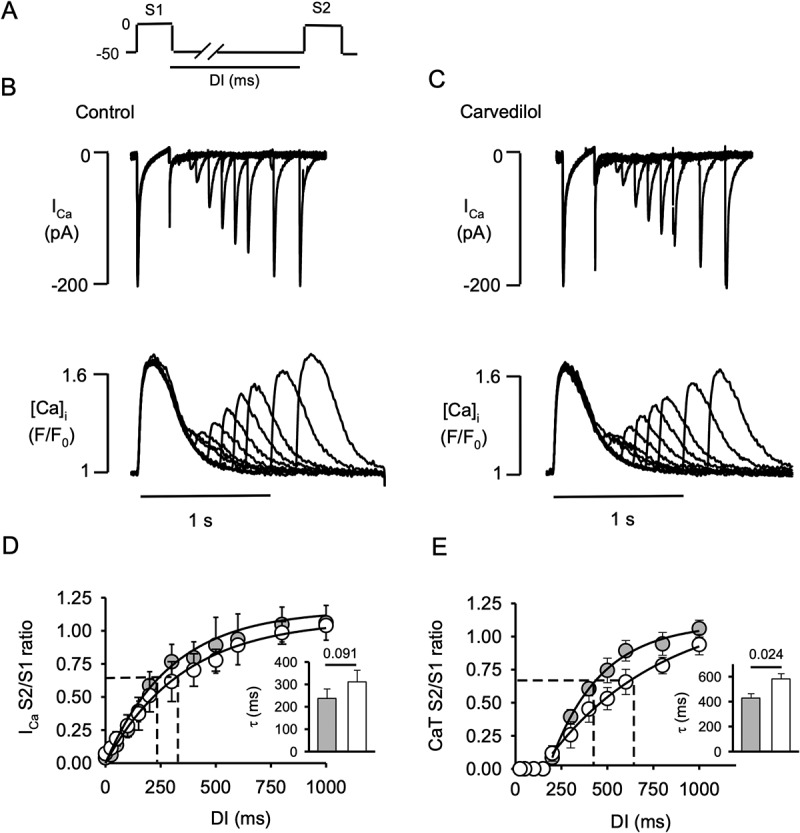
**Carvedilol prolongs the refractoriness of the SR calcium release**. A, Double pulse (S1-S2) protocol used to record Ca currents (I_Ca_) and CaT simultaneously in atrial myocytes. Series of S1-S2 stimulations were applied with progressively decreased diastolic intervals (DI) from 1,000 to 2 ms. B, Representative traces of I_Ca_ (top) and corresponding [Ca]_i_ profiles (bottom) in control. C, Representative traces of I_Ca_ (top) and corresponding [Ca]_i_ profiles (bottom) in presence of carvedilol (0.75 µM). D, I_Ca_ recovery from inactivation (RFI) curves in control (gray symbols) and in presence of carvedilol (open symbols). Dashed lines indicate time constant τ of I_Ca_ RFI kinetics derived from mono-exponential fit. Inset: average τ of I_Ca_ RFI (P = 0.091, n = 6, paired t-test). E, CaT and SR Ca release refractoriness in control (gray symbols) and carvedilol (open symbols). Inset: average time constant τ (mono-exponential fit) of CaT RFI (P = 0.024, n = 6, paired t-test).

I_Ca_ was monitored simultaneously with [Ca]_i_ in voltage clamp mode. To measure [Ca]_i_, atrial cells were loaded with Fluo-4 pentapotassium salt (100 µM) included in the patch pipette solution. Modified external Tyrode solution was composed of (in mM) 135 NaCl, 4 CsCl, 2 CaCl_2_, 1 MgCl_2_, 10 Hepes, 10 D-glucose; pH 7.4 with NaOH. The pipette solution contained (in mM): 130 Cs-glutamate, 20 CsCl, 0.33 MgCl_2_, 4 MgATP, 0.1 Fluo-4 pentapotassium salt, and 10 HEPES with pH adjusted to 7.2 with CsOH. To determine SR Ca release refractoriness and I_Ca_ recovery from inactivation (RFI) [Ca]_i_ and I_Ca_ were measured simultaneously using an S1-S2 protocol (Figure 2A). Single myocytes were held at −50 mV and depolarized to 0 mV for 250 ms (S1). A second 250 ms pulse to 0 mV (S2) was applied after DIs between 2 and 1,000 ms.

### Cytosolic [Ca]_i_ measurements

To monitor cytosolic CaTs atrial myocytes were loaded with the membrane-permeable Ca indicator Fluo-4 AM (Thermo Fisher Scientific, Waltham MA, USA) in non-patch clamp experiments, or Fluo-4 pentapotassium salt (Thermo Fisher Scientific) included in the pipette solution (see above). Fluo-4 AM (10 µM) loading occurred in the presence of 0.2% Pluronic F127 (Thermo Fisher Scientific) for 20 min and cells were subsequently washed twice for 10 min with standard Tyrode solution. Fluo-4 was excited at 488 nm (xenon arc lamp) and emission collected at >515 nm (photomultiplier tube). Fluo-4 [Ca]_i_ signals are presented as F/F_0_ ratios, where F_0_ represents diastolic [Ca]_i_. In non-patch clamp experiments, CaTs were elicited in standard Tyrode solution by electrical field stimulation using a pair of platinum electrodes (voltage set at ~50% above the threshold for triggering APs and CaTs, 5 ms pulse duration).

### APD and CaT alternans

**Figure 3.**
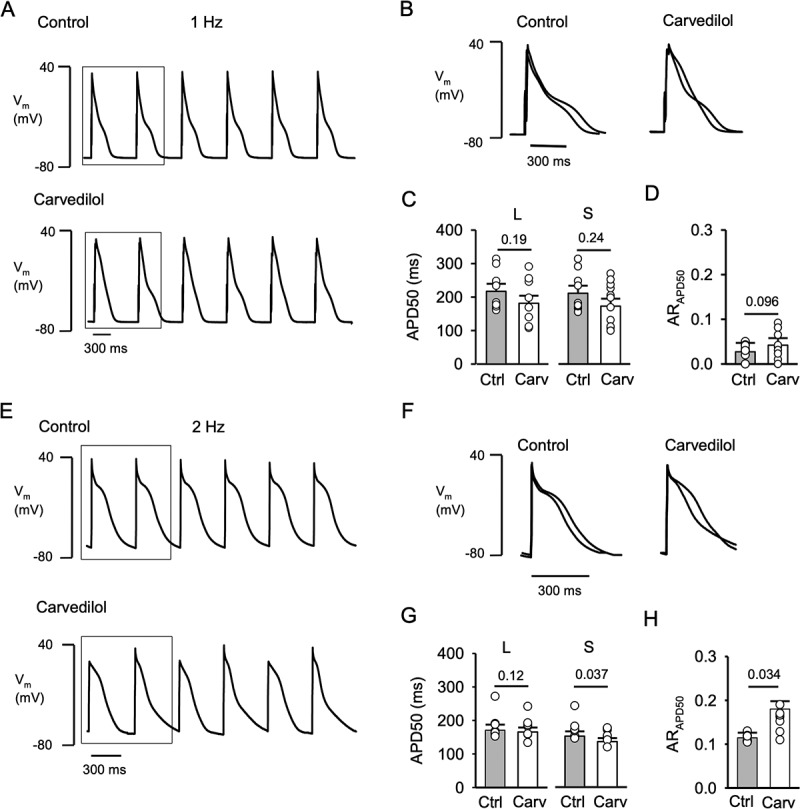
**Carvedilol induces APD alternans**. A, Representative AP traces recorded in control and in presence of carvedilol (0.75 µM) at 1 Hz. B, Overlay of 2 consecutive APs in control and carvedilol treated cells (marked by box in panel A). APs were recorded in current-clamp mode and evoked by 5 ms stimulation pulses of a magnitude ~1.5 times higher than AP activation threshold. C, APD of a pair of consecutive APs were classified as long (l) and short (s) based on APD50 analysis. Comparison of control and carvedilol mean APD50 of long (L: P = 0.19; n = 11) and short (S: P = 0.24) APs. D, AR_APD50_ in control and after carvedilol (P = 0.096). E, Representative AP traces recorded in control and in presence of carvedilol (0.75 µM) at 2 Hz. F, Overlay of 2 consecutive APs in control and carvedilol treated cells (marked by box in panel E). G, Comparison of control and carvedilol mean APD50 of long (L: P = 0.12; n = 9) and short (S: P = 0.037) APs. H, Average AR_APD50_ in control and after carvedilol treatment (P = 0.034). Statistical analysis with paired t-test.

CaT alternans was induced by incrementally increasing the pacing frequency until stable alternans was observed (Figure 1), or APD and CaT alternans were studied at fixed stimulation frequencies of 1 or 2 Hz (Figures 3, 5 and 6). The degree of CaT alternans was quantified as the alternans ratio (AR). AR = 1 – (CaT_Small_/CaT_Large_), where CaT_Large_ and CaT_Small_ are the amplitudes of the large and small amplitude CaTs of a pair of alternating CaTs. CaTs were considered alternating when CaT AR > 0.1. APD alternans were quantified at the 50% repolarization level (APD50) by APD50 alternans ratio AR_APD50_ = 1 – (APD50_short_/APD50_long_), where APD50_short_ and APD50_long_ are APD50 of short and long alternating APs.

**Figure 4.**
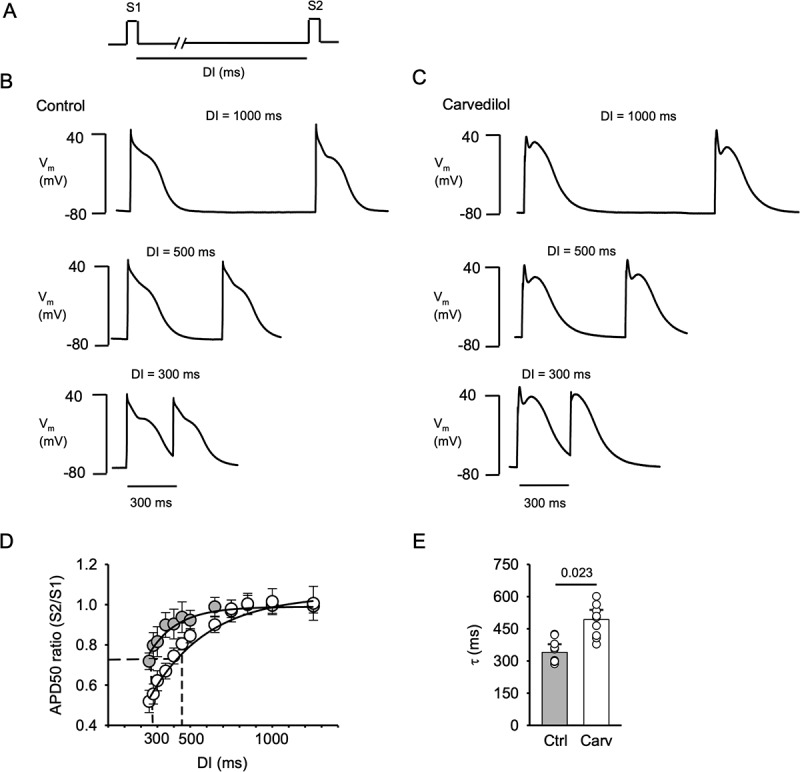
**Carvedilol prolongs AP restitution and steepens APD restitution curve**. A, AP restitution was investigated in current-clamp mode using a double pulse protocol (S1-S2). DI ranged from 250 to 1,250 ms. B, Representative AP traces recorded in control conditions at DIs of 1,000 ms, 500 ms and 300 ms. C, Representative AP traces recorded in presence of carvedilol (0.75 µM) at DIs of 1,000 ms, 500 ms and 300 ms. D, AP restitution curves. Mean APD50 S2/S1 ratios at DIs between 250 and 1,250 ms in control (gray symbols) and in presence of carvedilol (open symbols). Dashed lines indicate time constant τ derived from mono-exponential fit of the restitution curves. E, average τ of the restitution curves in control and after carvedilol treatment (P = 0.023, n = 9, paired t-test).

**Figure 5.**
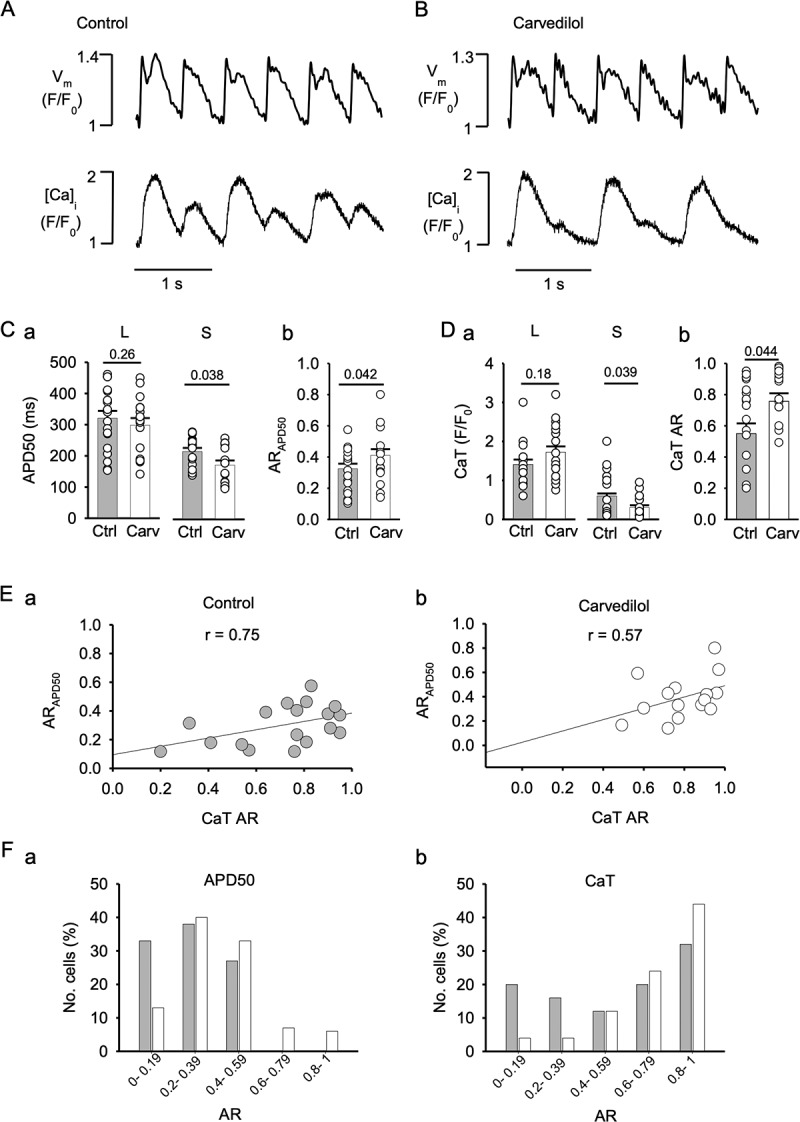
**Carvedilol increases APD50 and CaT alternans ratios**. Simultaneous AP and [Ca]_i_ recordings in control (A) and in presence of 0.75 µM carvedilol (B) using the fluorescent probes FluoVolt and Rhod-2. V_m_ and [Ca]_i_ signals were acquired in confocal transverse line scan mode. AP and CaT were elicited by field stimulation at 2 Hz. C, Mean APDs at 50% repolarization (APD50) of long (L) and short (S) APs (panel a) and AR_APD50_ (panel b) in control and in presence of carvedilol (n = 19). Statistical analysis with unpaired t-test. D, mean amplitudes of small (S) and large (L) alternans CaTs (panel a) and average CaT ARs (panel b) in control and in presence of carvedilol. E, Correlation between AR_APD50_ and CaT AR in control (a) and carvedilol (b) treated cells. r, correlation coefficient. F, distribution of AR_APD50_ (panel a) and CaT AR (panel b) in control (gray bars) and in presence of carvedilol (open bars).

**Figure 6.**
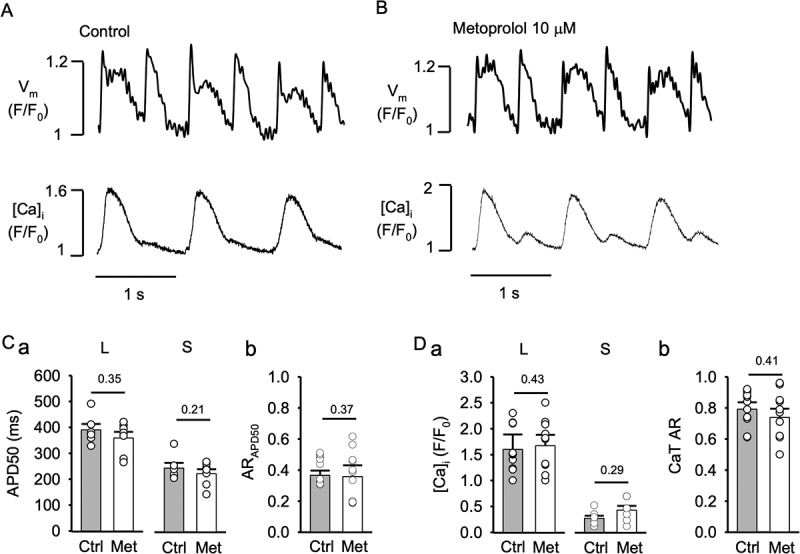
**Metoprolol does not modify APD or CaT alternans**. V_m_ and [Ca]_i_ traces from atrial myocytes in control (A) and during metoprolol (10 µM) treatment (B). APs and CaTs were elicited by field stimulation at 2 Hz. C, Mean APD50 of long (L; control vs. metoprolol: P = 0.35, n = 11) and short (S; P = 0.21) APs during alternans (panel a) and average AR_APD50_ (panel b) in control and metoprolol (Met; P = 0.37, n = 11). D, average CaT amplitudes of large (L: control vs. metoprolol, P = 0.43) and small (S; P = 0.29) amplitude alternans CaTs (panel a) and average CaT AR (panel b) in control and metoprolol (P = 0.41). Statistical analysis with unpaired t-test.

### Optical AP and [Ca]_i_ measurements

Simultaneous optical recordings of APs and [Ca]_i_ were conducted on atrial myocytes using the voltage-sensitive fluorescent probe FluoVolt and the membrane-permeable Ca indicator Rhod-2 AM (both Thermo Fisher Scientific). Confocal microscopy (Nikon A1R, Nikon Corporation, Melville, NY, USA) was used for APs and [Ca]_i_ imaging. Cells were loaded for 15 minutes with FluoVolt in standard Tyrode solution using proprietary (Thermo Fisher Scientific) loading conditions and dye concentration, followed by 10 min wash. Both FluoVolt^TM^ dye (component A of the Thermo Fisher loading kit) and FluoVolt^TM^ Loading Solution (component B) had a final concentration of 1X (specific concentrations not disclosed by manufacturer). FluoVolt preloaded atrial myocytes were subsequently loaded for 15 min with 5 μM Rhod-2 AM in the presence of 0.2% Pluronic F‐127. Cells were washed with Tyrode solution and preincubated with blebbistatin (10 µM) for 5 min to minimize motion artifacts. FluoVolt was excited at 488 nm and emission collected at wavelengths >515 nm, while Rhod-2 AM was excited at 543 nm and emission collected at wavelengths >600 nm. FluoVolt and Rhod-2 signals are presented as F/F_0_ ratios where F_0_ represents diastolic [Ca]_i_ during electrical pacing. [Ca]_i_ and optical AP measurements were conducted in the confocal line scan mode (512 lines/s) using a x40 oil-immersion objective lens. The scan line was placed along the transverse axis of the cell avoiding the nucleus. CaTs and optical APs were elicited by electrical field stimulation of intact atrial myocytes using a pair of platinum electrodes (5 ms voltage pulses set at ∼50% above the threshold for triggering APs and CaTs). Experiments were conducted at room temperature (22–24°C).

### Data analysis and presentation

Results are presented as individual observations and as means ± SEM, and n represents the number of individual cells. Statistical significance was evaluated using unpaired and paired Student’s t-test for single comparisons. Statistical analysis was conducted with SigmaPlot (v. 11; Systat Software). Differences were considered significant at *P* < 0.05. Monoexponential fits and time constants τ were calculated with SigmaPlot.

## Results

### Carvedilol facilitates CaT alternans

In the first set of experiments, we investigated whether carvedilol affects the inducibility and degree of CaT alternans in rabbit atrial myocytes. CaT alternans were elicited by electrical field stimulation and induced by incrementally increasing the pacing frequency until stable alternans was observed, usually between 1.5 and 2.5 Hz. After a control measurement, the same cell was subsequently exposed to carvedilol. Carvedilol was applied at concentrations not exceeding 1 µM because at such concentrations the aforementioned disruption of ECC was absent or minimal [15]. Carvedilol at 0.75 and 1 µM significantly increased CaT AR, while at 0.5 µM AR was not affected. Representative traces of CaT alternans in control and in the presence of carvedilol (0.5, 0.75 and 1 µM) are shown in Figure 1A and the average ARs are summarized in Figure 1C. In the presence of 0.75 µM carvedilol AR increased from 0.39 ± 0.06 to 0.51 ± 0.05 (P = 0.039, n = 11) and in 1 µM carvedilol AR increased from 0.34 ± 0.03 in control to 0.52 ± 0.04 (P = 0.001, n = 14). At 0.5 µM carvedilol no significant difference was found (control: 0.37 ± 0.03; carvedilol 0.35 ± 0.06; P = 0.31, n = 5). To test whether the effect of carvedilol on AR was potentially due to its well-known β-blocking action, the effect of the β-blocker metoprolol (10 µM) on AR was tested (Figure 1B). Metoprolol did not change the CaT AR (control: 0.42 ± 0.05; metoprolol: 0.37 ± 0.09, P = 0.41, n = 7), excluding the possibility that the carvedilol effect on AR was rooted in its β-blocking properties. Carvedilol also lowered the frequency threshold for pacing-induced CaT alternans. Atrial myocytes paced at 1 Hz showing no alternans were subsequently exposed to carvedilol (1 µM) which induced CaT alternans (Figure 1D) and increased AR from 0.03 ± 0.02 (no alternans) to 0.47 ± 0.14 (P = 0.008, n = 11; Figure 1E). Furthermore, we tested for the possibility that the AR changes in the presence of carvedilol were due to changes in SR Ca load (Figure 1F). We assessed the impact of carvedilol on SR Ca content by inducing SR Ca release with caffeine (10 mM). Steady alternans was recorded for 2 min before adding caffeine and the amplitude of the caffeine-induced CaT served to quantify SR Ca load. The SR Ca load was similar in control and in carvedilol treated atrial cells (Figure 1G; control: F/F_0_ = 3.31 ± 0.46; carvedilol: F/F_0_ = 3.17 ± 0.27; P = 0.46, n = 7). Taken together, the data indicate that the carvedilol effect on CaT alternans is not due to the β-blocker action or SR content alterations.

### Carvedilol prolongs refractoriness of SR calcium release

The refractoriness of RyRs and SR Ca release has profound effects on alternans susceptibility [5,17–23], and carvedilol is known to suppress RyR function and to enhance alternans in mouse hearts harboring a suppression-of-function RyR2 mutation (E4872Q) [23]. In the next set of experiments, we tested whether carvedilol (0.75 µM) also affected SR Ca release refractoriness and I_Ca_ RFI in native atrial myocytes. We included I_Ca_ in our study because it remains debated what role I_Ca_ plays in the generation of alternans and whether I_Ca_ alternates during CaT and APD alternans [19,24,25]. I_Ca_ and CaT were simultaneously recorded in voltage clamped atrial myocytes. V_m_ was kept at −50 mV (to inactivate I_Na_ and T-type Ca currents) and two depolarizing pulses (S1-S2) to 0 mV (250 ms duration) were applied. S2 was applied at different DIs ranging from 2 to 1,000 ms. The double pulse (S1-S2) protocol is shown in Figure 2A. Figure 2B (top) shows I_Ca_ elicited by S1 and by S2 at different DIs in control and in the presence of carvedilol (0.75 µM; Figure 2C). Figures 2B and 2C (bottom) show DI-dependent recovery of the CaT in control and in carvedilol. Analysis of amplitude and kinetic properties of the steady-state CaT (S_1_) revealed little effect of carvedilol on peak [Ca]_i_ (F/F_0_: control 1.44 ± 0.24, carvedilol 1.29 ± 0.19, P = 0.19, n = 6), rise time (RT), decay time (DT) and full duration at half maximum (FDHM) (RT: control 93 ± 8 ms, carvedilol 88 ± 8 ms, P = 0.35, n = 6; DT: control 434 ± 37 ms, carvedilol 395 ± 34 ms, P = 0.08; FDHM: control 299 ± 18 ms, carvedilol 310 ± 12 ms, P = 0.28). Figures 2D and 2E summarize RFI kinetics of I_Ca_ and CaT in control and carvedilol. RFI is expressed as ratios of I_Ca_ and CaT amplitudes elicited by the S2 pulse relative to the respective amplitudes elicited by S1 (S2/S1 ratio). S2/S1 ratios are plotted as a function of DI and fitted to a mono-exponential function and the time constants τ were compared. For I_Ca_ (Figure 2D), τ was 237 ± 46 ms in control and was not significantly different from τ after carvedilol treatment (311 ± 51 ms, P = 0.091, n = 6 insert in Figure 2D), however there was a tendency of carvedilol to slow RFI of I_Ca_. Overall recovery from inactivation of SR Ca release (Figure 2E) was approximately two-fold slower compared to I_Ca_. Carvedilol also slowed CaT RFI compared to control (control: τ = 428 ± 34 ms; carvedilol: τ = 582 ± 41; P = 0.024, n = 6). The faster kinetics of I_Ca_ RFI compared to CaT RFI suggests that the effect of carvedilol on beat-to-beat refractoriness of the entire ECC mechanism is dominated by an effect on the RyR and the SR Ca release machinery rather than I_Ca_ RFI.

### Carvedilol induces APD alternans

In general, CaT and APD alternans coincide; however, uncoupling between CaT and APD alternans has been observed [5–7,26,27]. After we established that carvedilol lowered the pacing threshold and enhanced CaT alternans dose-dependently (Figure 1), we addressed the effect of carvedilol on APD alternans. AP recordings were conducted in the current clamp mode and atrial cells were stimulated at 1 Hz (Figure 3A) and 2 Hz (Figure 3E). At 1 Hz carvedilol (0.75 µM) had no statistically significant effects on APD and AR_APD50_, however there was a tendency of carvedilol to shorten the AP (Figure 3C) and increase AR_APD50_ without reaching the criterion for alternans (AR_APD50_ > 0.1; Figure 3D). Examples of AP recordings in control and in carvedilol are shown in Figure 3A. Figure 3B shows overlays of two consecutive APs in control and in carvedilol. Figure 3C shows the averages of APD50 for long (L) and short (S) APs. The average APD50 for long APs in control was 217 ± 22 ms compared to 181 ± 24 ms in carvedilol (P = 0.19, n = 11), while APD50 of short APs was 211 ± 23 ms in control compared to 173 ± 28 ms in carvedilol (P = 0.24). At 1 Hz, the AR_APD50_ slightly increased from 0.03 ± 0.003 to 0.05 ± 0.005 in control and after carvedilol treatment, respectively (P = 0.096; Figure 3D), however remained below 0.1, thus the alternans threshold was not reached. While at 1 Hz carvedilol had no statistically significant effects on APD and AR, there was a tendency for AP shortening and increasing AR_APD50_.

Carvedilol effects on APD alternans were also tested at 2 Hz. For this analysis, only cells showing APD alternans, i.e. showing an AR_APD50_ > 0.1, were analyzed (from a total of 12 cells stimulated at 2 Hz 9 cells fulfilled the AR_APD50_ > 0.1 criterion). Figure 3E shows representative AP traces in control (top) and after carvedilol (bottom) recorded from the same atrial myocyte. Two consecutive superimposed APs in control and carvedilol are shown in Figure 3F. Carvedilol induced APD50 shortening. The mean APD50 of the long AP in control was 171 ± 6 ms and 165 ± 7 ms after carvedilol (P = 0.12, n = 9), and mean APD50 for the short AP changed from 152 ± 14 ms to 135 ± 4 ms (P = 0.037) after carvedilol (Figure 3G). Furthermore, at 2 Hz stimulation frequency, carvedilol increased AR_APD50_ from 0.11 ± 0.004 to 0.17 ± 0.011 (P = 0.034, paired measurements; Figure 3H). These data demonstrate that carvedilol shortened APD and enhanced the degree (AR_APD50_) of APD alternans.

### Carvedilol slows APD restitution

In the next set of experiments, using the S1-S2 protocol, we established the effect of carvedilol on AP restitution. Variations in AP restitution have been implicated in the genesis of APD alternans that can be arrhythmogenic [28–30]. In current clamp experiments, the double pulse (S1-S2) protocol was used with DIs ranging from 250 to 1,250 ms (Figure 4A). Examples of AP restitution in control (Figure 4B) and in the presence of carvedilol (Figure 4C) with DIs of 1,000 ms, 500 ms and 300 ms are shown. APD50 shortened with decreasing DI. Figure 4D shows APD50 restitution curves in control (gray symbols) and in carvedilol (open symbols). AP restitution curves represent APD50 elicited by S2 and normalized to APD50 elicited by S1 as a function of DI. Data were fitted to a mono-exponential function, and time constant τ was calculated. APD50 restitution was prolonged in the presence of carvedilol (0.75 µM) with τ increasing from 340 ± 28 ms to 493 ± 0.33 ms (P = 0.023 n = 6; Figure 4E). The slope of the APD restitution curves at DI = 500 ms, representing the stimulation frequency of 2 Hz that was used for alternans studies (Figures 3, 5 and 6), steepened in the presence of carvedilol and increased from 0.32 in control to 0.72 in carvedilol. While restitution of I_Ca_ was clearly faster (Figure 2D), AP (Figure 4E) and SR Ca release (Figure 2E) restitution kinetics were similar in control and similarly affected by carvedilol. These data suggest that AP and Ca release restitution are determinants of CaT and APD alternans and are similarly modulated by carvedilol.

### Correlation of APD and CaT AR

To further explore the carvedilol effect on CaT and APD alternans, a set of experiments was performed where APD and CaT alternans were measured simultaneously using fluorescent indicators. Atrial cells were loaded with the voltage sensitive fluorescent probe FluoVolt and the Ca indicator Rhod-2, and V_m_ and [Ca]_i_ were measured with confocal line scan imaging. APs and CaTs were elicited by field stimulation at 2 Hz. Representative traces of optical AP recordings from control (Figure 5A) and during carvedilol (0.75 µM) application (Figure 5B) and their corresponding [Ca]_i_ profiles show robust APD and CaT alternans that are more pronounced in the presence of carvedilol. Summary of mean APD50 in control (gray symbols) and after carvedilol (open symbols) for long (L) and short (S) APs are shown in Figure 5Ca. Mean APD50 for long APs in control was 320 ± 23 ms and 298 ± 24 ms (P = 0.26; n = 19) in carvedilol, and for short APs was 217 ± 11 ms in control and 173 ± 15 ms after treatment (P = 0.038). AR_APD50_ was 0.32 ± 0.03 in control and 0.41 ± 0.04 after carvedilol (P = 0.042) (Figure 5Cb). Figure 5D summarizes average small and large CaT amplitudes during alternans as well as CaT AR in control and in the presence of carvedilol. The large CaT amplitude (Figure 5Da) in control was F/F_0_ = 1.41 ± 0.12 and increased to 1.72 ± 0.15 in carvedilol (P = 0.18). The small CaT amplitude was 0.61 ± 0.07 in control and 0.31 ± 0.05 after carvedilol (P = 0.039). Carvedilol increased CaT AR (Figure 5Db) from 0.54 ± 0.05 in control to 0.75 ± 0.06 (P = 0.044). We calculated the strength of correlation of the linear relationship between AR_APD50_ and CaT AR. The correlation coefficient r for control conditions was 0.75 (Figure 5Ea) and 0.57 in the presence of carvedilol (Figure 5Eb). The effect of carvedilol on AR_APD50_ and CaT AR was further analyzed by binning the AR data in groups of 0.2 AR units (Figure 5F). Both the AR_APD50_ (Figure 5Fa) and CaT AR (Figure 5Fb) distributions are shifted to the right in cells treated with carvedilol, and AR_APD50_ extends over a broader range of AR values after treatment.

### The β-blocker metoprolol does not affect APD and CaT alternans

To determine if the effects of carvedilol on APD and CaT alternans are due to its β-adrenergic blocking action, we tested a different β-blocker. APs and CaTs were recorded simultaneously at 2 Hz using fluorescent probes in the presence of metoprolol (10 µM; Figures 6A and 6B). Metoprolol had no effect on APD and CaT alternans. Mean APD50 (Figure 6Ca) of long APs was 390 ± 24 ms in control and 359 ± 23 ms in metoprolol (P = 0.35, n = 11). Mean APD50 of short APs was 247 ± 21 ms in control vs. 226 ± 17 ms in metoprolol (P = 0.21), and AR_APD50_ values were essentially identical with AR_APD50_ = 0.36 ± 0.03 in control and 0.35 ± 0.07 after metoprolol (P = 0.37; Figure 6Cb). Similarly, metoprolol had little effect on CaT amplitudes (Figure 6Da; large CaT: F/F_0_ = 1.61 ± 0.29 in control vs. 1.67 ± 0.21 in metoprolol, P = 0.43; small CaT: F/F_0_ = 0.23 ± 0.04 in control vs. 0.37 ± 0.07 in metoprolol, P = 0.29) and CaT AR (0.79 ± 0.04 in control vs. 0.73 ± 0.05 in metoprolol (P = 0.41; Fig. 6Db). The data lent further support to the conclusion that the alternans effects of carvedilol were not mediated by its β-blocking properties.

## Discussion

In this study, we investigated the effect of the β-blocker carvedilol on pacing-induced CaT and APD alternans in rabbit atrial myocytes. The main findings are as follows: 1) carvedilol facilitates CaT and APD alternans, lowers the pacing frequency threshold of CaT alternans and increases the degree of CaT and APD alternans in a dose-dependent manner; 2) carvedilol causes CaT alternans by prolonging the SR Ca release refractoriness without changes in SR Ca load or I_Ca_ RFI; 3) carvedilol facilitates APD alternans by prolonging AP restitution and increasing the slope of the APD restitution curve; 4) the β-blocker metoprolol has no effect on CaT or APD alternans, demonstrating that the effects of carvedilol are not due to β-blocking action.

Carvedilol is a nonspecific β-blocker clinically used for the treatment of cardiovascular diseases, including hypertension, congestive heart failure, ventricular tachyarrhythmia, and atrial fibrillation [12,31–33]. Carvedilol has also α-blocking effects [14], can act as an antioxidant [34,35] and has anti-proliferative properties in vascular smooth muscle [36]. With respect to cellular Ca signaling, carvedilol has been reported to suppress RyR activity and spontaneous Ca waves [15], presumably by reducing the mean open time and, to a lesser extent, the P_o_ of the RyR Ca release channel [16].

Here, we report for the first time that carvedilol facilitates CaT and APD alternans in atrial myocytes (Figures 1, 3 and 5). At cellular and tissue level CaT and APD alternans largely correlate in onset and degree, however the correlation is not absolute. For example, we have shown previously that CaT alternans can develop in the complete absence of APD alternans [9]. In cardiac myocytes, the regulation of [Ca]_i_ and V_m_ is bi-directionally coupled ([Ca]_i_ ↔ V_m_ coupling) and governed by complex overlapping feedback pathways. It has been a longstanding and still ongoing debate about whether a disturbance of V_m_ or [Ca]_i_ regulation is the primary cause of alternans. V_m_-driven alternans (V_m_ → [Ca]_i_ coupling) is determined by APD restitution and activity of voltage-dependent ion channels that affect Ca signaling, whereas Ca-driven alternans ([Ca]_i_ → V_m_ coupling) stems from beat-to-beat disturbances of intracellular Ca cycling and affects V_m_ by altering Ca-dependent ion current and transporter activity. This bi-directional coupling is at the heart of the difficulties in establishing a mechanistic understanding of cardiac alternans and has been referred to as a classical “chicken or egg conundrum” in the literature [37,38]. While there is experimental and computational evidence for both, V_m_ → [Ca]_i_ [8,9,11,17,39,40] and [Ca]_i_ → V_m_ [4–7,19] coupling driven alternans, recent progress, including our own [9,19], and computational findings are increasingly pointing to Ca signaling disturbances as the primary cause of alternans [41–44].

### [Ca]_i_ → V_m_ coupling and Ca-driven alternans

With Ca-driven alternans, a disturbance of Ca signaling is the primary cause of CaT alternans and APD alternans is the consequence of CaT alternans. For example, in a previous study, we demonstrated that abolishing SR Ca release through RyR inhibition during simultaneous CaT and APD alternans normalized the AP and eliminated APD alternans, clearly demonstrating that CaT alternans triggers APD alternans [9]. In addition, studies using AP-clamped cardiac myocytes have demonstrated that APD alternans are not required for the development of CaT alternans [9,10,25,45]. Previously we have demonstrated that in field stimulated rabbit atrial myocytes during alternans the latency of cell-wide spontaneous Ca release after a triggered release was prolonged and Ca spark frequency was decreased after the large Ca transient [19]. Furthermore, during alternans, the recovery of SR Ca release, elicited by premature action potentials or by photolysis of caged Ca, was slower after a large-amplitude alternans CaT than after the small-amplitude CaT [19]. These findings indicate that SR Ca release refractoriness shows beat-to-beat alternations during CaT alternans and plays a key role in the generation of CaT alternans. CaT recovery depends on I_Ca_ recovery from inactivation, the SR Ca load, and the intrinsic properties of the RyR Ca release channel [19,46]. Here, we explored how carvedilol affects cellular Ca handling and how these changes could lead to an increase in alternans susceptibility.

Our results show that the mechanism through which carvedilol facilitated alternans was independent of I_Ca_ RFI. Carvedilol at the concentration used in our study only minimally slowed the kinetics of I_Ca_ RFI, and the difference was statistically not significant (Figure 2D). Carvedilol also minimally affected amplitude and CaT kinetics (rise and decay time, duration) arguing against the possibility that carvedilol had a major effect on Ca-dependent inactivation of I_Ca_. I_Ca_ RFI was much faster than recovery of SR Ca release and, thus, is not likely to play any significant role in the refractoriness of SR Ca release. In addition, carvedilol had no effect on SR Ca load (Figure 1G). Finally, we tested the effect of carvedilol on SR Ca release refractoriness. Several experimental and modeling studies [5,21,23,47–49], including our own experimental investigation [19], found that impairment of recovery of SR Ca release from inactivation is a strong causative factor underlying alternans. Here, we found that carvedilol prolonged SR Ca release refractoriness (Figure 2E). The kinetics of recovery of the CaT slowed by nearly 50% in the presence of carvedilol.

### V_m_ → [Ca]_i_ coupling and V_m_-driven alternans

It is generally agreed that in the case of V_m_-driven alternans a single parameter, the APD restitution slope, determines the development of alternans and controls alternans stability. Sustained APD alternans is critically dependent on diastolic interval (DI) and occurs when the slope of the APD restitution curve (i.e. APD as a function of DI) steepens and APD alternans risk is highest when the slope is >1 for a given stimulation frequency [4,22,28]. The AP is shaped by several membrane currents, including I_Ca_, I_Na_, NCX, Ca-activated Cl currents, and several K conductances. Several studies provided evidence that carvedilol affects I_Ca_ [14,15,50–52], I_Na_ [15,53,54], NCX [55] and K currents [50,56,57]. K current activity has been shown to modulate alternans susceptibility. In a previous study [10], we showed that activation of K_v11.1_ (also known as hERG channel, mediating rapid delayed rectifier K current, *I*_Kr_) and K_v7.1_ channels (*I*_Ks_, slow delayed rectifying K current) exerted protective effects from alternans through shortening of the action potential. Even though in our hands, the therapeutic concentration of carvedilol (0.5 µM) was not enough to induce ECC failures or statistically increase the AR in atrial myocytes, in this concentration range carvedilol inhibits transient outward K current (I_to_) [56] and the I_Kr_ [50] by 48.2% and 46%, respectively. K current inhibition causes APD prolongation, which in turn can facilitate alternans, but in the case of carvedilol this effect could be partially compensated for by a concomitant APD shortening due to I_Ca_ inhibition [15,50]. While it was not the scope of our study to dissect the exact contribution of these individual membrane currents to the carvedilol effect on alternans, we have shown in a previous study that carvedilol has an inhibitory effect on I_Ca_ and I_Na_ and leads to ECC failures at concentrations above 1 µM. Hence, I_Ca_ and I_Na_ inhibition by carvedilol putatively contributes to the slowing of APD restitution (Figure 4D/E). We also found a significant steepening of the APD restitution curve by carvedilol (the slope more than doubled) at the pacing frequency where our alternans experiments were conducted (2 Hz or DI = 500 ms) which constitutes a condition that facilitates V_m_-driven alternans.

### β-blocking agents and alternans

Clinically, the primary use of carvedilol is for its β-blocker action. β-adrenergic signaling is indeed involved in the development of alternans. We have shown previously that β-adrenergic stimulation rescued pacing-induced CaT and electro-mechanical alternans [24,58]. The results of these earlier studies indicated that in atrial myocytes, β-AR stimulation, acting via β_1_- and β_2_-adrenoceptors, regulates CaT alternans through parallel and complementary G-protein dependent signaling pathways that converge to rescue alternans. We concluded at the time that the redundancy in the β-AR signaling-mediated regulation of proarrhythmic alternans represents an inherent safety mechanism to prevent arrhythmogenic Ca release in the heart during enhanced sympathetic tone. Based on these earlier findings revealing profound effects of β-adrenergic signaling on alternans, we felt obligated to test whether the results observed in the present study could be explained by the β-blocking effects of carvedilol. However, we did not expect any effect of inhibiting β-adrenergic receptors because our experiments were conducted on isolated single cells in the absence of any extracellular neurocrine and paracrine agonists, i.e. in the absence of any basal β-AR tone. Nonetheless, we also studied the effect of an alternative β-blocker (metoprolol). As expected, metoprolol had no effect on CaT and APD alternans (Figures 1 and 6). In the presence of metoprolol, the duration of the long and short AP, the APD alternans ratio, amplitude of the small and the large alternans CaT, and the CaT AR were identical with control.

In clinical context β-adrenoceptor blockade with carvedilol has been reported to significantly reduce the frequency of premature ventricular contractions in patients with mild-to-moderate hypertension or chronic heart failure [59]. Although carvedilol has been shown to exert beneficial antiarrhythmic effects in patients with atrial fibrillation, it is important to keep in mind the potential adverse action resulting from CaT and APD alternans facilitation, although alternans facilitation by carvedilol only occurs at concentrations above therapeutical dosages (≥0.5 µM).

## Conclusions

In summary, we show here for the first time in atrial myocytes that carvedilol induces CaT and APD alternans. CaT alternans facilitation by carvedilol was due to a prolongation of the SR Ca release refractoriness, without changes in SR Ca load or I_Ca_ RFI. Moreover, prolongation of AP restitution and steepening of the APD restitution curve by carvedilol facilitates APD alternans. Within the conceptual framework of disturbances of the bi-directional coupling of [Ca]_i_ and V_m_ regulation as cause of alternans, our study revealed that separate disturbances of V_m_ and Ca signaling, induced by carvedilol, converge on generating conditions that favor alternans. Carvedilol through interference with ion currents directly relevant for AP generation (I_Ca_, I_Na_) leads to failure of ECC (shown in our previous study [15]), and, shown here, to a prolongation of APD restitution and steepening of the APD restitution curve, a hallmark sign of V_m_-driven alternans (V_m_ → [Ca]_i_ coupling). Carvedilol also significantly prolongs the refractoriness of the SR Ca release mechanism, presumably by acting on the RyR, consistent with the well-known effect of carvedilol decreasing the RyR open time and P_o_, and reducing RyR Ca release flux. Thus, carvedilol, by having different cellular targets, facilitates alternans in atrial myocytes through both V_m_ → [Ca]_i_ and [Ca]_i_ → V_m_ coupling, emphasizing the mutually dependent and entangled mechanisms of [Ca]_i_ and V_m_ regulation.

## Data Availability

The datasets analyzed in this study are available from the corresponding author on reasonable requests.
